# Researches on Phthisis

**Published:** 1836

**Authors:** M. Louis


Art. II.—Report made to the Royal Academy of Medicine, *on a Manuscript entitled*, "*Anatomico-pathological researches on Phthisis*, by M. Louis, of Paris." Translated for the Western Journal of Medical and Physical Sciences. By J. T. N. Maddox, M. D., of Louisville, Ky., and communicated through Professor Harrison.

You have charged us, M. M. Bourdois, Roger Collard and myself, to make you a report on a manuscript of M. Louis, entitled, "Anatomico-pathological researches on Phthisis." This subject has been handled so profoundly by Bayle, that he who attempts to write after him, on the same matter, will be, almost inevitably, the object of an unfavorable prejudice; this prejudice ceases however, when the circumstances which induced M. Louis to write, and the conclusions his observations have enabled him to make, are fully understood.

Arrived at the age at which most physicians discontinue their attendance at Hospitals, and cease to gather observations from any source, that they may devote themselves exclusively to practice, M. Louis has abandoned practice entirely, and devoted his time and attention exclusively to the study of facts.

Since October 1821, to the present period, July 12th, 1825, he has collected, with scrupulous care, the history of all the cases admitted in the wards St. John and St. Joseph, of the la Charite Hospital. The number of patients admitted was 1960, of which 358 died. Of these, 127 died of phthisis, and 40 others, who died of various maladies, had tubercles in the lungs. This lesion existed, then, in nearly the half of the deceased, and was in a third, the principal, if not the exclusive cause of death. The collection of a great number of facts, made for his own benefit, has furnished M. Louis with results sufficiently important to merit the attention of the Academy.

His work is divided into two parts: the first is devoted to the anatomical lesions, observed in the lungs and other organs; the second, to the exposition of the symptoms which indicate these lesions. The author has selected fifty cases, as a foundation for his work; and has added some observations relative to the general history, causes and duration of Phthisis.

It is only of tubercular Phthisis that he treats. He thinks, with M. Lænnec, that the granulations of Bayle are but tubercles in the formative stage, and that cancer, ulceration, melanoris, and calculi of the lungs should be referred to other kinds of disease.

First Part. The anatomical lesions of the lungs, have been so well described by Bayle and M. Lænnec, that the author confines himself to a very brief description, in which we find nevertheless, many remarks peculiar to him: for example, he remarked that tubercles not only affect, specially, the summit of the lungs, as they have said, but that when they exist in the divers lobes, those which occupy the summit are larger and more numerous, and that they soften at a period when those of the base are yet hard and comparatively undeveloped. He affirms, that differences relative to the number, size, and the more or less advanced state of the tubercles, apply more properly to the superior lobe compared with the inferior, than to the summit compared with the base. He has often seen the superior lobe entirely disorganized, while the corresponding parts of the inferior lobe, at the same height, were permeable to air and strown only with tubercles. In the course of his remarks, he gives two curious cases, in one of which a large excavation was filled with a fibrinous clot; in the other, a fragment of pulmonary tissue, in appearance perfectly healthy, was enclosed in a cavity, without any connection to the walls of that cavity.

The larynx, trachea, and bronchia have presented to M. Louis lesions very imperfectly described by Bayle. In one hundred cases, this physician found lesions in the larynx only in seventeen; according to him, ulcerations in the trachea are much more rare (he indicates no proportions); of lesions of the epiglottis, he says nothing. In one hundred and two cases, M. Louis found the epiglottis ulcerated eighteen, the larynx twenty-three, and the trachea thirty-one times. In many cases, he has seen ulcerations of the trachea occupy all its fleshy portion; and in one, many cartilaginous rings completely destroyed, in a part of their extent, by the progress of ulceration. The mucous membrane of the bronchia has oftenest presented no alteration in the vicinity of indolent tubercles, though it is found near excavations, particularly near those which are large, and are therefore considered ancient, to the thickened and reddened. He argues, thence, that the inflammation of the bronchial mucous membrane being posterior to the formation of tubercles, cannot be considered their cause, but rather the consequence of irritation produced by the matter of excavations. Inasmuch as the ulcerations in the trachea are large and more numerous posteriorly, and inasmuch as those of the epiglottis occupy almost exclusively its laryngeal surface, he infers that they are produced by the irritation of the expectorated matter.

In the tenth of the cases, the pulmonary parynchyma was the seat of an acute inflammation, supervening in the latter days of the disease.

The adhesions of the pleura, so common in Phthisis, has attracted particularly the attention of the author. He found but one case in which there were no adhesions. He observed generally a correspondence between the adhesions and the pulmonary disease: when the adhesions were feeble and limited, the excavations in the lungs were very small; when the adhesions were strong and extensive, vast cavities were found in the substance of the lungs.

He long since observed that tubercles are often developed in many organs at the same time, and that in consumptive patients, particularly, he often met with tubercles in other organs, as well as the lungs. Bayle had affirmed that tubercular matter sometimes exists in the intestines; but the numerical results which the work of M. Louis contains, was never presented before. He has recognized the presence of tubercles, or of tubercular matter in the small intestine, in nearly one-third of the cases. In the large intestine, in the ninth. In the mesenteric glands, in the fourth. In the cervical glands, in the tenth. In the lumbar glands, in the twelfth. In the prostate, in thethirteenth. In the spleen, in the fourteenth. In the ovaries, in the twentieth. In the kidneys, in the fortieth. In the womb, the cerebrum, the cerebellum, the spinal marrow, the uterus, once. These researches, made with unusual care, have conducted M. Louis to conclusions of vast importance. Out of three hundred and fifty-eight bodies he examined, he uniformly found tubercles in the lungs when they existed in any other organs whatever.

To this statement, even the tubercular depositions of the serous membranes in chronic inflammations, makes no exception. The attentive examination of the lungs, in all cases of this nature, has enabled M. Louis to perceive tubercles there. In one case only, in which death succeeded low fever, and in which the lungs appeared perfectly healthy, did he find some tubercular deposites in the mesenteric glands. Does this relation, observed in more than three hundred and fifty individuals, over fifteen years of age, obtain also in children? Numerous post-mortem examinations only can decide this matter.

The anatomical study of organs not specially the seat of Phthisis, furnished M. Louis with many additional facts of great interest.

There was increase of volume in the heart, only in three cases, and this augmentation was confined to the left ventricle. In the few instances where the heart was increased in thickness, the lesion existed almost always on the left side. These facts teach us the value of the theoretical speculations of authors, who consider the tubercular induration of the lungs as one of the most active causes of diseases of the heart in general, and of the right cavities in particular. In a majority of instances, M. Louis found the heart diminished in the same proportion as the other viscera. When measured, the aorta was found comparatively smaller, throughout its extent, in cases of Phthisis, than in those who died of acute affections. The same vessel was larger in cancerous, than in consumptive cases. In the fourth of the cases, it presented a remarkable redness of its internal face, without thickening or change in the consistence of its membranes. These lesions were not spoken of by Bayle, neither were those of the pharynx, of the osophagus, nor of the stomach, to which we have not yet referred.

Ulcerations of the pharynx and osophagus have been seen twice only. The osophagus presented, moreover, in three instances, attenuation, with softening of its membranes near its cardiac extremity. Ulcerations of these parts have not been seen by M. Louis in any other chronic malady: they have only been found in consumption and grave fever.

In ninety-six cases, in which the patient was carefully examined, M. Louis found the stomach nine times, double or tripple its ordinary size; the great curvature extending to the crests of the iliac bones. A like change in the size and position of the organ has only been seen in two instances of other diseases. But though this phenomenon is remarkable, the stomach offers, in its structure, lesions much more important, which were described in the author's article on "softening and attenuation of the mucous membrane of the stomach." This membrane, attentively examined in ninety-six cases, offered notable lesions in seventy-six, such as softening with attenuation, reddening with thickening, a nipple like aspect, &c.; in two cases it presented ulceration without any change of color or structure. This membrane while examined in comparison with nearly an equal number of cases of other chronic maladies, offered alterations in but half the cases; these alterations were likewise more inconsiderable; the softening and attenuation which was met with nineteen times, in ninety-six consumptive cases, has been seen but six times in eighty-four individuals, deceased from other chronic maladies. Thus the lesions of the stomach have been much more frequent and much more serious in cases of Phthisis.

The dueodenum was found ulcerated three times, and in some cases its mucous cryptæ enlarged.

The study of the anatomical lesions which are frequently seen in the small intestines, has induced M. Louis to fix his attention on the elliptical patches, formed by the agglommeration of the mucus cryptæ, of which some anatomists have spoken, but to which less importance has been attached than they merit. M. Louis believes that these patches, which exist in a healthy state, and are situated opposite the mesentery, participate but very little in the lesions of the mucus membrane which surrounds them, and are often the seat of lesions in which this membrane has no part. These patches are usually the seat of ulcerations in consumption.

M. Louis found those ulcerations in five-sixths of Phthisical cases, which proves that his examinations were more careful and minute than those of the diligent Bayle, who found them in two-thirds of the cases only. These ulcers have presented in their development this remarkable circumstance, that when they were confined to the mucous tunic, the subjacent cellular tissue was much thickened, and that when that was destroyed, the muscular membrane had undergone a proportionate increase of volume; so that while one of the tissues was being removed by ulceration, another was acquiring an increase of volume, and thus retarding an intestinal perforation. The mucus membrane presented, besides, some redness; it is rarely softened and thickened.

The large intestines presented ulcerations less frequently than the smaller intestines; but those ulcerations were larger. M. Louis has often seen the cœcum and the ascending colon ulcerated in all their extent. The softening of the mucus membrane is oftener seen there than in the small intestine: it exists in three-fourths of the cases.

The tubercular degeneration of the lymphatic glands of the mesentery has been seen, as we have said, in the fourth of the cases, all of which had ulcers in the intestines; but the tubercles were far from being in proportion to the size and number of the ulcers. In one case, in particular, where all the mesentric glands were tubercular, there was but one small ulcer

(a line in diameter) in the small intestine, the mucous membrane being, throughout its extent, every where else perfectly healthy. In many cases where the mesenteric glands were not tubercular, numerous ulcers existed in the bowels.

The author did not find a more constant relation between ulcers in the trachea and tubercular cervical glands. He therefore concludes that the tubercular degeneration of the lymphatic glands ought to be referred to other causes than inflammmation or ulceration of the adjoining membranes.

The fatty degeneration of the liver described by Bayle, the frequency of which has not been rigidly determined, is one of the most remarkable lesions which occurs as a consequence of Phthisis. It appears to be almost peculiar to this affection. M. Louis saw it in two-thirds of the cases of Phthisis, while in two hundred and twenty persons who died of other diseases, he has seen it but twice. He believes, also, that it is much more frequent in women than men, in the proportion of nine to ten. In the great majority of cases the duodenum was healthy. This fact is not uninteresting, since it has been affirmed that glandular affections are always consecutive to those of the neighboring membranes.

The spleen has often presented in this affection, as in others, alterations in volume and consistence. We have often seen them in the tubercular degeneration, as well as in the liver, the kidneys, the ureters, the prostate, the vesiculæ seminales, the ovaries and the uterus.

The peritoneum presented traces of recent inflammation in four cases. In another were found miliary semi-transparent granulations. In one case, two folds of this membrane, known under the designation of epiploon and mesentery, were formed of tuberculous, greyish semi-transparent matter, imperfectly mixed, and had acquired upwards of an inch in thickness.

Remarkable lesions were formed also in the brain. In three-fourths of the cases, the lateral ventricles contained from one to three table spoons of serosity, and the sub-arachnoidal tissue of the superior part of the brain was infiltrated.

In five cases the brain was softened throughout; in five others, the softening was partial, pulpy, and confined to the centre. The collection of small granulations that is generally found at the summit of the brain, on either side of the longitudinal sinus, and which are of a glandular nature, according to some anatomists, should be considered morbid productions in the opinion of M. Louis. He bases this opinion, firstly, on their entire absence in many individuals; secondly, on the inequality they present in their development, together with the thickened and opaque appearance the arachnoid around them constantly assumes.

This, the first part of M. Louis' work is terminated with an investigation into the peculiar lesions of Phthisis, and those which should be regarded as wholly adventitious. He refers to the first class adhesions of the pluera, more particularly in the upper part of the chest, ulcers in the air and digestive passages, the fatty degeneration of the liver and tubercles in any part of the body; to the second, peripneumony, acute pleurisy, ramolissement of the mucus membrane of the stomach and bowels, peritonitis, arachnitis and ramolissement of the brain.

Second Part. After having given a succint description of Phthisis, in conformity to his observations of facts, the author examines particularly some of the principal symptoms; designates the period of their accessions, their frequency, and some other circumstances of which authors hitherto have spoken very vaguely.

Hæmoptysis, with which he commences is present in two-thirds of Phthisical cases: in a fifth it appeared before the cough and spitting. Of the nineteen hundred and sixty cases, under the author's observation, none had hæmoptysis but consumptive patients, except a few, in which it was the consequence of external violence, from a contusion of the parietes of the chest, a fall, and a small number of females laboring under a suppression of the menses. From this the author concludes, that with the specified exceptions, the existence of an abundant hæmoptysis renders very probable the presence of tubercles in the lungs. Your commissioners believe that this terrible annunciation is happily invalidated, at least partially, by many other facts. Hæmoptysis is more frequently met with in women than in men, in the proportion of three to two.

The pains in the chest which consumptive patients experience, may either be referred to pulmonary tubercles, or pleural adhesions; and as these two species of lesion usually accompany each other, it is difficult to determine by opening the body, which produces the pain. The acute sensibility of the pleura, and the well established absence of pain in a great number of tubercular cases, external and internal, induce us to believe that this phenomenon has its seat in the exterior membrane of the lungs, rather than in their parenchymatous structure. Nevertheless, it is not always so: a woman, whose case is reported in the work, complained during the fourteen days which preceded her death, of pains between the shoulders; post-mortem examination discovered only encysted tumors without adhesion, and besides, all the patients in which these adhesions were confined to the summit of the lungs had not complained, during life, of pains in the chest.

The examination of the febrile phenomena which supervene in all cases of Phthisis, in the second period of the disease, presented the following results. The chills have occurred in five-sixths of the cases; the sweats in nine-tenths. The opinion promulged by the ancients, and reiterated by the moderns, that the night-sweats and diarrhœa alternate, is in opposition to the observations of M. Louis, not only in Phthisis, but in other maladies. The author has seen the diarrhœa diminish sometimes when the night-sweats appeared, but it soon resumed its original intensity, without a subsidence of that symptom. M. Louis here remarks, quite appropriately, ins peaking of prolonged and abundant sweats, that a function can for a long time be profoundly altered, without an appreciable change, to our senses, of the organ which has charge of it.

Diarrhœa was present in every case, save one. When it preceded death a short time only, from four to twenty days for example, he found ordinarily very small ulcers in the intestines, the mucus membrane softened, often reddened and thickened. When the diarrhœa was of long standing, there were almost always vast and numerous ulcerations in one or the other intestine, sometimes in both. The diarrhœa was not less violent when the ulcers were confined to the small intestine principally, than when they were more extensive in the large intestine: a fact not uninteresting at this period, when some systematic physicians are disposed to make the colon the exclusive seat of lesions originating that symptom.

The symptoms which characterize ramollissement of the stomach—perforation of the lung, and pneumo-thorax its sequent, of which the author has given some cases—ulcerations of the epiglottis, of the larynx and trachea—are objects of special examination. We shall not speak of symptoms furnished by softening of the stomach and perforation of the lung, on which subjects M. Louis has presented particular treatises; we shall only speak of the other lesions.

The subjects in which the epiglottis was ulcerated, complained of pain, on a line with or above the summit of the thyroid cartilage, of difficulty of deglutition, sometimes so great that liquid returned by the nose, a local pain more or less acute, and complete aphonia for one, or many months. Ulcerations of the trachea, with a few exceptions, have given, ordinarily, no peculiar symptoms.

Lesions of the stomach have been accompanied by symptoms which will enable us in future, to recognize their presence during life. In a great number of cases, inappetency, epigastric pain, and even nausea and vomiting have indicated these lesions. With regard to vomiting, all physicians have considered hitherto, that it is provoked by the cough exclusively. M. Louis believes that this may be the case in the beginning of the disease, but observes that under those circumstances, patients should be free from epigastric pain, have a good appetite, and digest their food with facility, during the intervals of the coughing paroxysms. By observing these symptoms, we may readily distinguish sympathetic vomiting from that which is ascribable to lesions of the mucus membrane of the stomach.

Nausea being a common symptom in Phthisis, and Phthisis being a common disease, it was both easy and interesting to know what relation existed between the state of the tongue during life, and that of the stomach after death, that we may the better appreciate the value of assertions always made on this subject. Of ninety-six cases in which the stomach has been examined after death, most minutely, this viscus has appeared healthy in nineteen cases; in the remaining seventy-seven it has presented various lesions. Of the nineteen cases in which it had a healthy aspect, nine had had during life a tongue more or less red. In one of these last, the redness and dryness of the tongue was very great, persisted from the entrance of the patient, into the hospital to his death, thirty-two days afterwards, and nevertheless, the stomach, in all respects, was perfectly healthy. Among the seventy-seven others, in which the stomach offered, either a simple softening with attenuation of the mucus membrane, or a redness more or less extensive, with or without nipple-like projections, etc., only thirty-five had had the tongue red during life, and in five of them an inconsiderable redness only existed. It appears, therefore, that the redness of the tongue has been as often seen in cases where the stomach was healthy, as in those where this organ had undergone "grave" changes.

The diagnosis of Phthisis is easy at an advanced period; the world knows well that it is uncertain during the first stage, and sometimes for a very long period. M. Louis has endeavored particularly to determine, by the comparison of facts and observations, what signs should make us first suspect and then recognize the presence of this disease. A dry cough of long standing, a shortness of breath induced by walking or speaking, pains more or less acute in the back or in the sides of the chest, a notable diminution of flesh and strength, should cause us to suspect strongly the existence of tubercles in the lungs; should one or several attacks of hæmoptysis supervene, the presence of Phthisis can scarcely be doubted. Percussion and auscultation then will dissipate any doubts , if the sound of the chest be obscure below one of the clavicles to an inconsiderable extent; if the respiratory murmur is heard there more fully and is accompanied by a rale; these two phenomena existing in this part only, where tubercles are usually first developed, their presence can no longer be questioned. M. Louis reported a case, in which, by the aid of these signs, the presence of the disease was recognized seveenteen days after the appearance of the first symptoms.

In the second period, when tubercles are succeeded by excavations, the diagnosis is generally easy; pectoriloquy furnishes an unerring evidence. We should, however, remember that the partial dilatation of some of the bronchia can give rise to this phenomena, and therefore call in requisition all the other signs and assistances which are calculated to confirm us in our opinions. An interesting case is cited as the basis of this very sage precept.

M. Louis presents in other articles, interesting extracts, relative to the duration of the malady, the circumstances under which we most frequently meet with it, and sudden deaths which are not uncommon in Phthisis, of the cause of which post-mortem examinations do not always furnish adequate notions. Relating to its duration, he remarked that a greater number of women died in the first year, in proportion, than men. This he ascribes to the greater frequency of secondary lesions, as the fatty degeneration of the liver and the ramolissement of the mucus membrane of the stomach, which he has met with much more frequently in females.

Two articles are devoted to latent and to acute Phthisis. Five cases have been seen by M. Louis, in which fifty days did not elapse between the appearance of the first symptoms and the death of the patients. Death occurred in one instance as early as the twenty-fourth day.

Finally, in a last chapter, M. Louis examined by the aid of facts, the question of the nature of tubercles, a question so often and so uselessly discussed by the aid of reasoning and induction, and concluded that the tubercular degeneration of the lungs, neither was of the nature, nor consequence of inflammation, but essentially different from both.

In this analysis we have aimed to present you only the principal results, to which the author's investigations have brought him.

We think the Academy, specially charged with giving a good direction to the labors of physicians, cannot too much encourage the number, always too small, of those who, in place of abandoning themselves with the crowd to systematic discussions, dedicate their lives to the collection of facts at the bed side of the sick; and to seeking, after death, the traces of disease which have preceded and lead to it, and to deduct from the collation of these facts rigorous consequences.

(Signed) BOURDOIS,
ROGER COLLARD,
CHOMEL, (*Reporter.*)

REMARKS OF THE TRANSLATOR.

When we see a man of such discipline and acuteness of mind as Louis, such zeal in the pursuits of science, such freedom from prejudice, such comprehensiveness of intellect, and such self-denying disinterestedness, leaving a valuable private practice, for nearly four years, entering a hospital and subjecting himself to all the inconveniences of that situation, in order that he may the better understand the nature and tendency of a disease; we feel confident that medicine must be materially benefited; and the profession redeemed from the imputation which ignorance and charlatanism have brought upon it. That ours is not a "system of scientific guessing," is apparent to all acquainted with its principles and practice; and that the science of medicine is susceptible of the utmost precision and accuracy, at least so far as diagnosis is concerned, cannot be questioned, even by the superficial observer.

This is particularly the case with diseases of the chest, the recognition and diagnosis of which, till within a few years, has been so little understood. Louis' work does him great credit, and is honorable to the profession, of which he is so distinguished a member. To be properly estimated it must be studied diligently. Such devotees to the best interests of humanity, such benefactors to poor, frail, fallen mortality, must occupy a large space in the gratitude of nations, and purchase for themselves an unfading wreath of medical honors.

To practitioners, no fact is more important than that the upper part of the lungs is much more liable to tubercular degeneration than any other. It often happens, that while this portion of the lung or lungs is filled with tubercles, and even with abscesses, the inferior lobe or lobes of the same lung are comparatively free from disease. This is so uniformly the case, that nothing, probably, but accident ever causes tubercular matter to be developed primarily in the inferior lobes. We are indebted for this most satisfactory observation to the illustrious Louis, who met with but two exceptions in one hundred and twenty-three cases that he carefully examined.

The superior lobe of the left lung is most usually the location of this most intractable disease. Sometimes the right lung is affected, the left being entirely free from disease. Occasionally both are involved. Dr. Morton in his valuable and highly creditable "Illustrations of Pulmonary Consumption," gives the following as the result of his observation:

| | | | | | | |
|---|---|---|---|---|---|---|
| "Right lung diseased most in | | | | | 28 | cases. |
| Left | do | do | do | in | 51 | do. |
| Both lungs equally diseased in | | | | | 7 | do." |

Of the preceding number, the disease was confined to the left lung in four cases, and to the right lung in a single instance only.

Until the time of Louis, the attention of the profession was not directed to any part of the chest, as the seat of tubercular degeneration. Since he has announced that the summit of the lungs is, almost invariably, the first to undergo this morbid change, pathologists have endeavored (perhaps vainly) to explain the fact. They affirm that motion in this part of the chest is more limited than in any other, in consequence of the fixedness of the first rib; that in this part of the lungs, the circulation of the blood meets with most difficulties, and therefore, that lesions of nutrition are most likely to occur at this point, but we are not satisfied with this explanation, which seems to us too mechanical.

The difficulty of distinguishing Phthisis from chronic Bronchitis, and other pectoral affections, has been hitherto considered almost insurmountable. To form opinions, physicians were compelled to wander in the fields of hypothesis; and their conclusions were but the offspring of conjecture. But now we know, that the ravages of Phthisis are confined almost to the superior lobes, and we know, equally well, that pneumonic inflammation is confined almost exclusively to the inferior lobes of the lungs. By the aid of physical signs, we are now able to dispel or confirm the fears of those who suffer from pectoral affections.

Some pathologists believe that tubercles are the result of chronic inflammation. Inflammation of the lungs is a very common consequence of this disease, especially of its advanced stages, when the cavities are large, and when their proximity to the pleura is so great, that they act as irritants to this membrane; but that the morbid action which leads to the deposition of tubercular matter, is essentially different from inflammation, must be apparent to all who are familiar with post-mortem appearances. We find the pulmonary tissue even in contact with tubercles, perfectly healthy, presenting no traces of pneumonic inflammation, which would not be the case, were each tubercle the focus of inflammatory action.

Dr. Carswell considers the bronchial mucus membrane the most common seat of tubercles, in which opinion Dr. Clark of London concurs. Andràl and others believe that the pulmonary cellular tissue is the seat of this affection. The latter opinion is more popular with our ablest pathologists.

That family peculiarities are transmissible from parent to progeny, has been known for ages. To persons of vigorous minds and bodies, this must be a most delightful contemplation; but to the gouty, the consumptive, the scrofulous, and the maniacal, in wedded life, this must be a subject of gloomy apprehension, and physicians should regard it a solemn duty to warn all who are predisposed to these maladies against marriage.

When a predisposition to consumption exists, very little irregularity on the part of the indvidual will favor tubercular development. Predisposed persons should, therefore, avoid any and every irregularity, and pay the strictest attention to diet, cleanliness, and exercise in the open air. Impure air, ardent spirits, tight lacing, neglect of cleanliness, unwholesome food, the inhalation of irritating vapors, and particles of matter, sedentary habits, imperfect clothing, neglected catarrhs, the depressing passions, and whatever tends to debilitate the constitution, or disturb the nutritive functions, may act as exciting causes in predisposed persons. Nay more, by a habitual disregard of health, and a daily indulgence in, or neglect of, the above specifications, this terrific malady may be acquired even in individuals without predisposition; for if the deposition of tubercular matter depends on derangement of the nutritive functions, it must be obvious, that whatever tends to produce that derangement, may give rise to the formation of tubercles.

Phthisis prevails in all classes of society, and in persons of almost every temperament, and variety of bodily conformation. From time immemorial, it has been supposed, that persons of light hair, fair skin, blue eyes, a narrow chest, and a soft and delicate expression of countenance, were most liable to attacks of consumption. In this country, though these peculiarities greatly predominate, we find that this opinion of authors is inconsistent with truth. On the contrary, nearly two-thirds of Dr. Morton's cases occurred in persons of dark hair, dark eyes and dark complections. In my observation this disease has attacked persons without regard to temperament or expression of countenance.

Authors have endeavored to identify Scrofula and Phthisis, and have boldly affirmed that "Phthisis is Scrofula of the lungs." Their arguments on this subject are vague and unsatisfactory. We are disposed to regard them as dogmas of the day, received without strict investigation. Cases are on record in which scrofula made frightful ravages in the osseous and muscular tissues, and even terminated in death, nevertheless no tubercles were found though they were diligently sought after. Besides we often see consumptive persons who never had a symptom of scrofula. In addition to this, it is not uncommon to see the bronchial glands free from disease in persons who have died of Phthisis. If scrofula and consumption be diseases of the same kind, is it likely that scrofulous caries of the spine should successively pass through all its stages, and even end in death, without causing tubercular depositions in the lungs? Would not the frail consumptive suffer more from scrofulous enlargements of the cervical glands? and especially should not the bronchial glands be the seat of scrofulous disease in consumptive cases?

Hæmoptysis has always been considered an alarming symptom by practitioners; because they regarded it as the beginning of a most unmanageable disease. Louis tell us that "a fifth of his consumptive cases were ushered in by this symptom, and that of the nineteen hundred and sixty cases which came under his observation, none were troubled with hæmoptysis but consumptive persons; except a few from external violence, amenorrhea, &c., &c.: the committee think, however, that this statement is at least partially invalidated." Louis believes that the abundant hæmoptysis is a proof of the existence of tubercles, from which opinion other eminent pathologists dissent. It would be contrary to all analogy to believe, that the pulmonary mucus membrane is not sometimes the source of hæmorrhage: and, besides, it is evident, that under certain circumstances, violent muscular exertions may produce such a congestion of the lungs, that nothing but a copious hæmoptysis, from the parenchyma of the lungs, (save the intervention of professional aid,) can rescue the patient from danger. Rare instances are given, in which, copious hemorrhage from the lungs resulted from the rupture of a large vessel, by violent muscular effort; and also of abundant hæmoptysis from the parieties of tubercular excavations. Hæmorrhages from the two latter causes, are, however, comparatively rare. "M. Andral believes hæmoptysis to be at once a proof and consequence of pulmonary congestion, a state which he considers necessary to the formation of tubercles; and having also on several occasions found, both in man and in the horse, tubercles deposited in a coagulum of blood in the lungs, he concludes, that in this way the effusion of blood may become an exciting cause."*

Hectic fever is regarded by some as a symptom of Phthisis. In its appearance it is usually tardy and insidious. In a majority of instances it is a part of the sad drama, which deprives the world of so much youthful loveliness, talent, and beauty. In degree, it is by no means uniform, it being more modified by collateral and accidental complications than any other symptom. Gastric and intestinal irritations, and inflammatory affections of the respiratory organs, are the complications which have so much influence in exciting and modifying this fever—an influence which, according to some, is even greater than the tubercular affection.

Hectic, like intermittent fever, has its cold, and hot, and sweating stages, which symptoms usually attend every paroxysm. Its accession is commonly in the afternoon, from which time it continues till midnight, when it terminates in a profuse and exhausting perspiration. These paroxysms usually occur every day; in some cases they recur twice daily.

Instead of a regular hectic paroxysm, we sometimes have only a dry and burning sensation in the palms of the hands, and in the soles of the feet, in some instances confined to the

* Clark's Treatise on Pulmonary Consumption, p. 188.

former, in others to the latter; which is a source of considerable inconvenience. Some consumptive patients are almost constantly troubled with a distressing species of febrile excitation.

J. McM., aged 30, a laborer, of healthy parents, was admitted in the Louisville alms-house, August 15th, 1835. He then had ägue and fever, accompanied with cough and dyspnœa. His life had been irregular—rather dissipated. For the last two years he has suffered much from cold, and dyspnœa, prior to which he was rarely troubled in this way. Since his admission his cough has been constant, though treated actively with antimonials, etc. He has had no symptom of hæmoptysis. Bowels exceedingly regular, notwithstanding the liberal employment of opiates, for the cough.

February 15th, 1836. I ascertained by means of the stethescope, the existence of a cavity in the middle lobe of the right lung (which proved to be in the inferior portion of the superior lobe). During his illness he has had, on four or five occasions, copious nocturnal perspirations, and as many as three alvine dejections daily. His perspiration usually possesses an acid odor. On one occasion it commenced about sunrise, was abundant, and continued about two and a half hours. The action of the heart has been remarkably calm and normal, rarely exceeding its natural standard either in force or frequency.

March 28. Respiration principally abdominal. Pectoriloquy in both lobes of left lung. Percussion discovers an evident dullness on the left side of the chest, which we attribute to induration of the left lung. Expectoration, amounting to a pint daily; is purulent, tenacious, pelotonnated. Conceiving his case to be irremediable, I have used palliative remedies only, for the last two months, in the composition of which opium entered largely, and yet his bowels were never so regular.

29. Acute pain in the left side, aggravated by coughing. Expectorated blood several times, twice, as much as ℥ss, pulse 76.

April 2. Pulse 72. Some œdema of the feet.

9. Nine alvine dejections last night. Pulse 96.

12. Expectorated and vomited about three pints of matter last night, which he supposes is from his right lung.

21. Two attacks of hæmoptysis; discharged in each about ℥vj. arterial blood.

May 8. Dyspnœa. For several days has complained of costiveness.

June 2. Died at 6½ o'clock, A. M. Latterly injections were necessary, on several occasions, to obviate costiveness. With two exceptions, his pulse has been uniformly calm, numbering about 72 strokes per minute. On one occasion it exceeded 100. The nocturnal perspirations occurred at such long intervals as rarely to require attention. Had fistula in ano of two or three years standing. Body emaciated to the last degree.

Dissection, six hours after death.

Left Lung.—Adhesions numerous, firm, and extensive in superior lobe. In the inferior lobe they were less numerous, and easily broken up. Traces of recent inflammation coextensive with its pleural envelope. Both lobes indurated, and intimately adherent in consequence of pleural inflammation. Superior lobe filled with anfractuous cavities, anteriorly superficial. Grey and red induration of the inferior lobe.

Right Lung.—Costal and pulmonic pleura every where adherent. A cavity as large as a hen's egg in the lower portion of the superior lobe. The balance of this lobe filled with tubercles. A part of the middle lobe greenish and indurated. Lower lobe crepitant, gorged with blood, and strown with tubercles.

Stomach, a little enlarged, otherwise healthy.

A blush of redness only in the lower portion of the ilium.

Remarks. This we conceive to be an interesting case, because we regard it as one of acquired consumption: neither of his parents having had any symptoms of Phthisis. It is interesting, because it shows that the lungs may be nearly destroyed, and yet the patient suffer very little from abdominal derangement. This man had neither nausea nor vomiting; rarely diarrhœa, and usually possessed a good appetite. It is interesting, because of the almost complete absence of hectic fever, and nocturnal perspirations. It is interesting because it shows that three-fourths of the lungs may be rendered useless by the extension of disease, and nevertheless the heart exhibit no evidences of embarrassed action. I met not long since a physician who affirmed that his patient had consumption because her pulse numbered more than a hundred strokes per minute. We know not what he would have said of our patient had he seen him.

One of the most distressing complications of Phthisis is, diarrhœa. Commencing early in the disease, it sometimes continues throughout its whole course. Louis reports but one case in which it was absent. He concludes that diarrhœa is the result of intestinal ulceration, and that it will be more or less obstinate and troublesome, in proportion to the size and number of the ulcers. As these ulcers enlarged and deepened, the subjacent membrane is often hypertrophied apparently to prevent the consequences of perforation.

Diarrhœa, at all times debilitating, is particularly so to one harassed by a continued cough, enervated by protracted disease and sleepless nights, and especially so to one whose lungs are no longer capable of performing their part in the transformation of chyle into blood. It is consequently a matter of the utmost importance to meet promptly the first appearances of this exhausting complication: indeed it is the duty of practitioners to prevent it if possible. For this purpose, the diet of consumptive patients should be restricted to the mildest and most digestible articles of food, and they should be admonished to avoid carefully those indulgencies and irregularities which are calculated to excite abnormal actions in the intestinal canal. Irritation of the mucus membrane of the bowels, when once established, is soon followed by inflammation, ulceration, and consequently diarrhœa, which, in conjunction with extensive pulmonary disease, rapidly exhausts the vigor of the system, and hastens the period of dissolution.

The influence of age, of sex, race, climate, and condition in the development of tuberculous disease is very imperfectly understood. We know quite as little about the influence of occupation and the various modes of living. It is highly important that the profession be well informed in these particulars, and it is to be hoped that no time will be lost in making this enquiry with that care and diligence which the subject so richly merits, and which is so imperatively demanded by the interests of science and the world. The co-operation of numerous observers, at different points, in different climates, embracing every variety of condition and mode of living, is necessary, to furnish satisfactory information on a subject of so much utility,—with which the happiness of the human family is so closely identified.

The period of infancy is by no means exempt from tubercular disease. "Chaussier discovered miliary tubercles in the lungs of a fœtus, which died at birth, and an encysted abscess, or rather vomica, in the lungs of another." "Husson reported to the Paris Academy of Medicine the case of a still-born babe, at the seventh month of pregnancy, which had tubercles in the lungs in a state of suppuration." "M. Lombard believes, that tubercles are present in one-eighth of the children, who die in the Hospital des Enfans Malady, at Paris, between the ages of one and two years." We should, however, remember that "Velpeau and Buschet never observed tubercles in the fatal state, in the course of their observations, and M. Guizot did not find a single example of tuberculous diseases in four hundred new-born children, whom he examined."*

"More than one-fourth $\left(\frac{27}{100}\right)$ of those who die, from birth to puberty, are affected with tuberculous disease; yet it causes death in about one-sixth of the cases only."†

*Clark's Treatise on Pulmonary Consumption, p. 130.

†Ibid, p. 134.

The following tables contain all the facts we possess on this subject; though the number is small, they are highly interesting. We take them from Dr. Clark's Treatise already referred to. We are encouraged to hope that when our professional brethren see this paucity of facts, on which we have to make our calculations, that they will resolutely determine to obviate this difficulty. It would require little additional time to note the necessary and interesting facts in each case. Were a few facts contributed, by each physician, the period would soon arrive, when we should have ample materials, on which all our arguments and deductions might rest securely. The day of reasoning and speculation is past. The profession need facts, not speculative theory.

### Table I.

| Age. | Total Deaths. | Tuberculous. | Non-Tuberculous. | Tuberculous in 100 Deaths. |
|---|---|---|---|---|
| 1 | 2630 | not ascer'd. | not ascer'd. | not ascertained. |
| 2 | 1290 | 161* | 1129* | 12* |
| 3 | 729 | 292 | 437 | 40 |
| 4 | 408 | 204 | 204 | 50 |
| 5 | 263 | 173 | 90 | 66 |
| 6 | 178 | 130 | 48 | 72 |
| 7 | 125 | 87 | 38 | 70 |
| 8 | 99 | 74 | 25 | 75 |
| 9 | 82 | 52 | 30 | 63 |
| 10 | 78 | 52 | 26 | 67 |
| 11 | 77 | 44 | 33 | 57 |
| 12 | 78 | 47 | 31 | 60 |
| 13 | 80 | 60 | 20 | 75 |
| 14 | 84 | 56 | 28 | 66 |
| 15 | 89 | 47 | 42 | 52 |

* We have not sufficient data to establish the frequency of tubercles during the first two years of life.

### Table V.

Showing the mortality from Consumption above the age of fifteen years.

| Place of Observation. | 15 to 20 | 20 to 25 | 25 to 30 | 30 to 35 | 35 to 40 | 40 to 45 | 45 to 50 | 50 to 55 | 55 to 60 | Above 60 |
|---|---|---|---|---|---|---|---|---|---|---|
| 1. Edinburgh, | 6 | 9 | 13 | 8 | 11 | 8 | 6 | 3 | 9 | 4 |
| 2. Berlin, | 18 | 28 | 27 | 27 | 39 | 29 | 20 | 32 | 39 | 53 |
| 3. Nottingham, | 42 | 73 | 76 | 46 | 51 | 28 | 20 | 11 | 5 | 6 |
| 4. Philadelphia | 182 | 974 | | 875 | | 565 | | 338 | | 258 |
| 5. Chester, | 15 | 27 | | 24 | | 22 | | 16 | | 6 |
| 6. Carlisle, | 15 | 45 | | 34 | | 31 | | 15 | | 15 |
| 7. Paris--Louis | 11 | 39 | | 33 | | 23 | | 12 | | 5 |
| 8. do.—Bayle | 10 | 23 | | 23 | | 21 | | 16 | | 4 |
| 9. Charleston, | | 26 | | 24 | | 13 | | 21 | | 3 |
| 10. do.—whites | | 14 | | 17 | | 10 | | 3 | | 3 |
| 11. do.—blacks | | 15 | | 13 | | 9 | | 3 | | 3 |

These facts show, conclusively, the importance of attending to the physical education of children in the strictest manner.

Hippocrates, the father of medicine, designated the period of life, between the eighteenth and thirty-fifth year, as that in which death from Phthisis is most frequent. The correctness of this observation has been fully established by modern pathologists.

The influence of sex over this disease is not known. According to some authors, more males than females die of Phthisis. Others believe that it is most prevalent among females. Perhaps a majority of authors incline to the latter opinion.

Dr. Morton, of Philadelphia, reports a case (13) in which, notwithstanding extensive tuberculous deposition in both lungs, and a vomica as large as a hen's egg in the superior and posterior portion of the lobe of the left lung, there was none of the usual symptoms of Phthisis. "Louis mentions a case in which there was neither cough nor expectoration, though there was hectic and hæmoptysis: both lungs were tuberculous, and one of them had a cavity."

From our own observation we are induced to believe, that there are a vast number of erroneous notions, in the profession, concerning this disease. Many physicians speak of it so vaguely, that we are convinced they know little or nothing of its real character. The importance of a correct discrimination of this, from other pulmonary affections, must be apparent to all; and the criminality of ignorance in such momentous concerns must be equally obvious. We should bear in mind that Phthisis has no peculiar symptom. Any or all of its usual manifestations may be absent, though the disease be making excavations, and filling the parenchymatory structure of both lungs, with tuberculous matter, such being the case, we cannot too earnestly call the attention of the profession to a subject, fraught with such momentous consequences to society, to science, and to generations yet unborn.

But for the introduction of physical signs and assistances, physicians would now experience the same difficulties, in recognising particular diseases of the chest, which tended so much to embarrass and unsettle our predecessors from the time of Hippocrates. The discovery of percussion by Avenbruggen, is one of the most valuable means of diagnosis, in many diseases, with which our profession has ever been enriched. It renders the diagnosis of diseases, once obscure and difficult, much more easy and certain; and gives, consequently, to the practitioner a power to which he was formerly a stranger. But though our science has been greatly enriched by the introduction of percussion, this means of diagnosis will not compare in importance, with auscultation, which was practically introduced to the notice of the profession, by the immortal Laennec. The utility of this invention can only be appreciated by him who is conversant with its advantages. In many affections of the heart, in Phthisis, in Pleurisy, its agency is indispensable, if an accurate knowledge of the patient's situation is necessary. With the aid of these two diagnostic means, a physician's uncertainty and hesitation, particularly in thoracick diseases, are dispelled with the same facility that the mists of a summer morn are dispelled by the glorious sun of day. Since their introduction, the accuracy of our science has wonderfully increased, and it must continue to advance till the nature of every species of thoracick affection is perfectly understood. For the following valuable observations, I am indebted to that accurate observer and diligent physician, Dr. Morton, of Philadelphia. His work comprises so much useful information, in a small compass, that that physician will do himself and his patients injustice who fails to read it carefully. The work is worthy of a place in every medical library.

"Clothing.—Human caprice and extravagance are in nothing more remarkably shown than in dress: experience, disease, even death itself, convey unheeded monitions to those who yield an idolatrous homage to fashion. I am aware that on this subject it is almost in vain to expostulate; but if the people have no regard for themselves, they ought at least to indulge some for their offspring, and I submit the following remarks."

"Whenever the surface of the body is kept so cold as to produce uneasiness and distress, the circulation of the blood then is diminished, and the internal and more delicate organs are proportionably overloaded and oppressed. This inequality, in a vital process, cannot be long kept up with impunity: according as it is sudden or repeated, or protracted, it is capable of inducing the most afflictive diseases to which the human frame is liable—rheumatism, catarrh, inflammatory affections of the viscera, scrofula in scrofulous constitutions, consumption in those predisposed to it, etc. In fact, it may be safely asserted, that the integrity of no one of the organic functions is more essential to health than that of the skin: and I am convinced, after much observation, that a large number of the consumptions that occur between the ages of eighteen and thirty years, might have been prevented by a proper attention to dress in childhood. No infatuation is more preposterous than that which is familiarly called the *hardening* of children, by exposing them half clothed, to every vicissitude of temperature, and by subjecting them to injudicious cold bathing. These practices are peculiarly objectionable in a climate so inconstant as ours: and I have no hesitation in saying, that where one constitution is invigorated by them, twenty are absolutely destroyed.

"What is true of prevention in childhood, is of equal application in the therapeutic treatment of adults. In vain is the use of medicine, or the regulation of diet: in vain are all the other precautions that ingenuity can devise; if the skin is not kept warm, and its healthy secretion maintained by proper attention to the quantity and quality of clothing. As winter approaches, the chest of the invalid should be coated in flannel up to the neck, and the same dress should be extended down the arms to the wrists: and where this material is insufficient to prevent the sensible access of cold, a buckskin vest ought to be worn over it. The body and lower limbs are to be protected in like manner, and particular attention given to the feet; for if the latter are habitually cold, the whole system will participate in the inconvenience."

"It is well known to those who have given attention to this subject, that even Russia, with its intense cold, is a far less consumptive climate than England; and the difference is solely attributable to the Russian custom of keeping their houses warm, clothing themselves in furs, and taking particular care to preserve their feet from cold and damp."*

"In Holland, which is in the same latitude with England, consumption is rare among the native population, and for the very reasons which prove an exemption in Russia."

"I could mention several examples, both in children and adults, in whom the constitution has been suddenly and effectually restored, from a languid and almost hectic condition, to comparatively robust health, by a timely change of dress in the manner above mentioned; and I must, once for all, repeat, that without this precaution, all other measures, whether prophylactic or remedial, will end in disappointment."

"Exercise.—It is obvious from what has been said in the

* Dr. Reid on Consumption, p. 200. The lower classes of people in St. Petersburg suffer much more from Phthisis than the wealthy, owing to the absence of household comforts.

preceding section, that clothing modifies the climate in which we live in a most important manner: thus we may feel the vicissitudes of temperature, or not, according as we expose or protect the surface of the body: for under the latter circumstances, there are but few days in the year in which the lungs, even in this climate, cannot bear the cold of winter without inconvenience. I therefore entirely coincide in the views of my friend and preceptor, Dr. Parrish,* that 'vigorous exercise, and free exposure to the air, are by far the most efficient remedies in pulmonary consumption. It is not, however, that kind of exercise usually prescribed for invalids,—an occasional walk or ride in pleasant weather, with strict confinement in the intervals,—from which much good is to be expected. Daily, and long continued riding on horse-back, or in carriages over rough roads is, perhaps, the best mode of exercise; but when this cannot be commanded, unremitted exertion, of almost any kind in the open air, amounting even to labor, will be highly beneficial. Nor should the weather be scrupulously studied. Though I would not advise a patient to expose himself recklessly to the severest inclemencies of the weather, I would nevertheless warn him against allowing the dread of taking cold, to confine him on every occasion, when the temperature may be low, or the skies overcast." Fatigue should be generally avoided.

By the foregoing translation and remarks, we hope to succeed in calling the special attention of the profession, to a disease, which existed in "nearly one-half" of those who died at the la Charite Hospital,† and in more than a fourth of the children who died before puberty;‡ to disease which spares neither age, sex, race, nor condition; to a disease which causes us to mourn, almost daily, over the early decease of youthful promise and loveliness; to a disease which, alas! often nips the bud of life in childhood's hour, and rings with anguish the parental bosom. If we shall have contributed to the better understanding of the pathology and means of preventing a disease, which numbers among its victims a Bayle

*North American Medical and Surgical Journal, for 1829 and 1830.
†Louis. ‡Clarke.

and a Lawrence; if we shall have succeeded in inducing abler minds to inquire into the causes, nature, means of prevention, and treatment of Phthisis; we shall feel amply compensated for our trouble in preparing this article. We feel that the article is imperfect. We regret that it is so. Our hope, however, is, to excite inquiry; to remove erroneous opinions; to prevent the administration of repeated doses of medicine and confinement to hot bed rooms, when the patient should be invigorated by exercise in the open air; and to awaken the minds of all concerned to the consequences of insufficient clothing at every period of life: in childhood, more especially. The infrequency of Phthisis, in the cold climate of Russia, and in Holland, is a fact of no little interest to the profession. The cause should be diligently inquired into; and if it be found to be referable to peculiarity of dress, or that insufficiency of clothing materially assists in the origination of Phthisis, then, indeed, may we rejoice, that a disease, "styled the opprobrium of physic," is entirely managable, provided, those concerned heed the monitions of enlightened pathology.

